# Switching Separation Migration Order by Switching Electrokinetic Regime in Electrokinetic Microsystems

**DOI:** 10.3390/bios14030119

**Published:** 2024-02-22

**Authors:** Alaleh Vaghef-Koodehi, Blanca H. Lapizco-Encinas

**Affiliations:** Microscale Bioseparations Laboratory, Biomedical Engineering Department, Rochester Institute of Technology, 160 Lomb Memorial Drive, Rochester, NY 14623, USA; av1462@g.rit.edu

**Keywords:** electrophoresis, elution order, microfluidics, microparticles, separation

## Abstract

Analyte migration order is a major aspect in all migration-based analytical separations methods. Presented here is the manipulation of the migration order of microparticles in an insulator-based electrokinetic separation. Three distinct particle mixtures were studied: a binary mixture of particles with similar electrical charge and different sizes, and two tertiary mixtures of particles of distinct sizes. Each one of the particle mixtures was separated twice, the first separation was performed under low voltage (linear electrokinetic regime) and the second separation was performed under high voltage (nonlinear electrokinetic regime). Linear electrophoresis, which discriminates particles by charge, is the dominant electrokinetic effect in the linear regime; while nonlinear electrophoresis, which discriminates particles by size and shape, is the dominant electrokinetic effect in the nonlinear regime. The separation results obtained with the three particle mixtures illustrated that particle elution order can be changed by switching from the linear electrokinetic regime to the nonlinear electrokinetic regime. Also, in all cases, better separation performances in terms of separation resolution (*Rs*) were obtained by employing the nonlinear electrokinetic regime allowing nonlinear electrophoresis to be the discriminatory electrokinetic mechanism. These findings could be applied to analyze complex samples containing bioparticles of interest within the micron size range. This is the first report where particle elution order is altered in an iEK system.

## 1. Introduction

Analytical separation techniques are essential in the identification, quantification, and purification of chemical and biological components. Differential migration techniques are able to separate samples into their components by exploiting differences in the migration velocity of analytes within a separation column, capillary or channel. Chromatography and electrophoresis are perhaps the most widely used differential migration techniques, both of which have several distinct application modes, as both are well-stablished techniques supported by significant developments reported over the last few decades [1].

In all migration-based separation methods, the migration order (also called elution order) of the analytes being separated is a major aspect of aspect of the separation process. There are cases where a specific elution order of the analytes is required to obtain a better quantification of a minor component in mixture [2] or to elute first a fragile component. In many electrokinetic (EK) separation methods, such as CE, significant efforts have been dedicated to manipulating the migration order of analytes in a separation process. In the majority of EK systems electroosmotic flow (EOF) is present and usually employed as liquid pumping mechanism, however, in contract with electrophoresis (EP), EOF is a non-separative transport. Thus, strategies for inhibiting, reversing or suppressing EOF have also been established, which vary from dynamic, static, and static absorbed coating agents [3].

The numerous reports in the field of chiral separations with capillary electrophoresis (CE) are excellent examples of applications where the elution order of analytes, called enantiomer migration order (EMO), can be altered by adjusting the system’s conditions. Chiral CE is the separation of enantiomeric pairs, which are identical molecules that only differ in the spatial arrangement of their atoms or functional group [4]. For these separations, a chiral selector is added to the background electrolyte (BGE), which binds reversibly with the enantiomers, altering their apparent mobilities. Thus, the separation process is significantly influenced by the concentration and binding constant of the chiral selector [5]. Several strategies have been reported in chiral CE for manipulating the EMO, including the use of counterbalancing pressure and modification of the apparent mobilities and binding constants achieved by altering the composition of the BGE and concentration of the chiral selector [4].

Another example of EK techniques where elution order has been carefully altered or reversed, is the separation of histones (proteins that provide structure to chromosomes) by modifying BGE composition [6]. Reverse migration-micellar electrokinetic chromatography (RM-MEKC) is a novel EK method where migration of analytes is heavily manipulated. In this method negative voltages are employed in addition to reduced EOF to force analytes with a positive charge to migrate towards the cathode. This arrangement allows micelles that carry the negative charged analytes to migrate towards the anode. This results in two phases migrating in opposite directions that meet at a specific unction location for analytes to mix and enhance detection sensitivity [7].

While altering elution order has been demonstrated with chiral CE, histone CE, and RM-MEKC for identical molecules and proteins, there remains a necessity to explore the possibility of altering the elution order of micron-sized particles such as microorganisms and mammalian cells in EK systems [8,9,10,11,12,13]. This study presents the first manipulation of the migration order of analytes (microparticles) in an insulator-based electrokinetic (iEK) system stimulated with a DC potential. A novel strategy was employed in this work that allows the separation process to be switched from the linear to the nonlinear EK regime. In the former linear electrophoresis (EP_L_) is the dominant mechanism, while in the latter nonlinear electrophoresis (EP_NL_) is the dominant mechanism [14,15]. Linear electrophoresis, the EK phenomenon that enabled the numerous developments in CE reported during the 20th century [14], is an excellent method for separation analytes by exploiting differences in their electric charge, but EP_L_ cannot differentiate analytes by size or shape [16,17]. Nonlinear electrophoresis, which has received significant attention recently [14,15], can differentiate analytes by their size and shape [14,18,19]. Until recently, the majority of iEK systems stimulated with DC or low-frequency AC signals had ignored the effects of EP_NL_. It was believed that dielectrophoresis (DEP) was the dominant phenomenon in these systems, and thus, inaccurate interpretations of experimental results were reported and numerical models required the use of correction factors to match experimental results [20]. Recent reports [21,22] from several groups unveiled the dominant effects of EP_NL_ in iEK systems which had been first reported by Dukhin since the 1970’s [23], but the lack of experimental data hindered its widespread application [24].

The present study reports the separation of three distinct mixtures of polystyrene microparticles: a binary mixture of two types of microparticles with almost same electrical charge but distinct diameter (5.9 and 11.7 µm), and two tertiary mixtures of particles of distinct sizes (2.4 to 11.7 µm) and differing electrical charges. Particle zeta potential is the parameter employed in this work to assess electrical charge. Each particle mixture was separated twice; the first separation was carried out under a low DC voltage to enable a charge-based separation under the linear EK regime, and a second separation process under higher voltage to enable a size-based separation under the nonlinear EK regime. The voltages employed in these separations were sufficient to reach the moderate electric field regime, where the velocity of EP_NL_ has a cubic dependence with the electric field. The new knowledge of EP_NL_ enables novel separation strategies, where the elution order of the microparticles being separated can be modified by simply switching the EK regime of the separation. The findings from this study illustrate that iEK systems can be fine-tuned in the same manner as well-known techniques such as chiral CE, histone CE or RM-MEKC. The new developments in the field of EK can enable new separations strategies, opening new possibilities for the separation of micron-sized particles, including microorganisms. This is the first report where elution order is altered in an iEK system by leveraging the new knowledge on the phenomenon of EP_NL_. The findings from this work have the potential to be utilized in the analysis of bioparticles.

## 2. Theory

**Figure 1:**
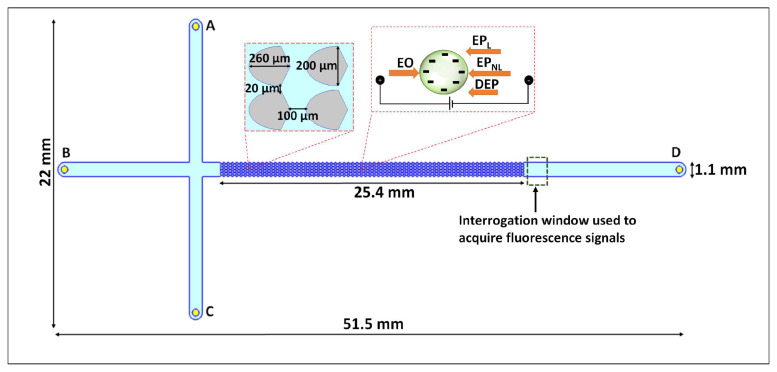
Illustration of the iEK PDMS microchannel with asymmetric insulating posts employed in this study. The channels have a standard cross-T with four liquid reservoirs labeled A–D. The left inset depicts the dimensions of the asymmetric insulating posts. The right inset shows the four EK forces (EO, EPL, EPNL, and DEP) acting on the particles.

The EK phenomena considered in this study are electroosmosis (EO), linear and nonlinear EP and DEP. Thus, the overall velocity ($v_{P}$) of a particle in the iEK device depicted in Figure 1 is described by the following expression:

(1) $$v_{P}=v_{EO}+v_{EP,L}+{v_{DEP}+v}_{EP,NL}^{\left(n\right)}$$

where $v_{EO}$, $v_{EP,L}$, $v_{EP,NL}^{\left(n\right)}$, and $v_{DEP}$ are the EO flow velocity, the linear and nonlinear EP velocities and the DEP velocity, respectively. It is common to classify EK phenomena with respect to their dependence with the electric field (E). The velocity expressions for two linear EK considered here are:

(2) $$v_{EO}=\mu_{EO}E=-\frac{\varepsilon_{m}\zeta_{W}}{\eta }E$$

(3) $$v_{EP,L}=\mu_{EP,L}E=\frac{\varepsilon_{m}\zeta_{P}}{\eta }E,\;\mathrm{known}\;\mathrm{as}\;\mathrm{the}\;\mathrm{weak}\;\mathrm{field}\;\mathrm{regime}\;$$

where the mobilities of EO flow and EP_L_ are represented by $\mu_{EO}$ and $\mu_{EP,L}$, respectively; and $\varepsilon_{m}$ and η denote are the medium permittivity and viscosity, respectively, the zeta potential of the channel wall and particle are $\zeta_{W}$ and $\zeta_{P}$, respectively. The velocity expressions of the two nonlinear EK phenomena, that is, the phenomena that do not depend linearly on E, are [18,25]:

(4) $$v_{EP,NL}^{\left(3\right)}=\mu_{EP,NL}^{\left(3\right)}E^{3},\;\mathrm{known}\;\mathrm{as}\;\mathrm{the}\;\mathrm{moderate}\;\mathrm{field}\;\mathrm{regime},\;\mathrm{which}\;\mathrm{occurs}\;\mathrm{at}\;\beta \;\leq \;1,\;\mathrm{arbitrary}\;\mathrm{Du},\;\mathrm{and}\;\mathrm{Pe}<<1$$

(5) $$v_{EP,NL}^{\left(3/2\right)}=\mu_{EP,NL}^{\left(3/2\right)}E^{3/2},\;\mathrm{known}\;\mathrm{as}\;\mathrm{the}\;\mathrm{strong}\;\mathrm{field}\;\mathrm{regime},\;\mathrm{which}\;\mathrm{occurs}\;\mathrm{at}\;\mathrm{for}\;\beta >1,\;\mathrm{Du}\;<<\;1,\;\mathrm{and}\;\mathrm{Pe}>>\;1$$

(6) $$v_{DEP}=\mu_{DEP}\nabla E_{rms}^{2}=\frac{r_{p}^{2}\varepsilon_{m}}{3\eta }Re\left[f_{CM}\right]{\nabla E}^{2}$$

where $\mu_{EP,NL}^{\left(n\right)}$ is the mobility of the EP_NL_ velocity, and *n* represents the electric field dependence. As shown in Equations (4) and (5), analytical expressions for the moderate and strong regimes exist, which are the limiting cases of low and high Peclet number (*Pe*). The parameter β represents the dimensionless applied field strength coefficient and *Du* is the Dukhin number [14,21,26,27]. Under the experimental conditions employed in this work, all microparticles followed the moderate electric field regime (See Table S1 in the Supplementary Materials). The expressions used for estimating these three dimensionless parameters are included in the Supplementary Materials. The DEP velocity expression is illustrated in Equation (6), where $r_{p}$ is the particle radius; $Re\left[f_{CM}\right]$ is the real part of the Clausius-Mossotti factor which accounts for polarization effects and ${\nabla E}^{2}$ is the gradient of the squared magnitude of E.

The quality of the experimental separations was assessed employing the parameter of separation resolution (*Rs*), which is estimated directly from the electropherograms, employing the particle experimental retention time ($t_{R,\;e}$) and the peak width (W) at the peak base. The expressions for *Rs* is:

(7) $$Rs=\frac{2(t_{R2,\;e}-t_{R1,e})}{W_{1}+W_{2}}$$

## 3. Materials and Methods

### 3.1. Microfluidic Devices

Microfluidic channels were fabricated from polydimethylsiloxane (PDMS, Dow Corning, MI, USA) employing standard cast-molding techniques [8]. Each channel was sealed employing a glass wafer that was coated with PDMS, to ensure that all interior channel walls had the same properties. Each PDMS device was employed for a maximum of 5 days to ensure the stability of wall zeta potential [28]. Figure 1 contains an illustration of the microchannel which had a standard cross-T channel design to allow for EK sample injection. Channel dimensions are included in Figure 1 along with two insets depicting the insulating posts dimensions and an illustration of the EK forces acting on the particles. Not shown in the image is the channel depth which was 40 µm. Asymmetrical insulating posts (oval-diamond) shapes were selected as this configurations has shown to be highly effective enhancing the discriminatory capabilities of the devices [8].

### 3.2. Microparticle Samples and Suspending Media

Six distinct types of polystyrene microparticles (Magsphere, Pasadena, CA, USA and Spherotech, Lake Forest, IL, USA) were employed in this study, and their characteristics are listed in Table 1, all of them possessed a negative surface charge. Particle suspensions for experimentation were created by diluting stock suspension into the suspending media which was a 0.2 mM K_2_HPO_4_ solution with the addition of 0.05% (*v*/*v*) Tween 20 to prevent particle clumping. The media had a conductivity of 39.2 ± 3.1 µS/cm pH of 7.3 ± 0.9. This media produced a $\zeta_{W}$ = −60.1 ± 3.7 mV in the PDMS channel walls and a EO flow mobility of $\mu_{EO}=4.7\pm 0.3\times 10^{-8}\;m^{2}V^{-1}s^{-1}$, as measured in our laboratory employing current monitoring experiments.

### 3.3. Experimental Procedures and Equipment Information

Prior to each experimental session, microdevices were soaked for 12–14 h with the suspending medium to ensure stable EO flow. A sample of 5–10 µL of the selected particle suspension was added to reservoir A, then electric potentials were applied employing a high voltage power supply (Model HVS6000D, LabSmith, Livermore, CA, USA). The applied potentials were used for both, sample EK injection and also to perform the iEK particle separation process. The magnitude of the electric fields listed in Table 2 is not high enough to cause Joule heating in the iEK channel, as established by previous study [29]. The EK injection process consisted of three steps: loading, gating and injection, as listed in Table 2. All experiments were visualized and recorded employing a Leica DMi8 (Wetzlar, Germany) inverted microscope equipped with a color camera.

## 4. Results and Discussion

Three distinct microparticle separations were performed, a binary separation and two tertiary separations, to illustrate the use of EP_NL_ to alter the elution order of the microparticles in the separation. The voltage conditions employed to carry out the EK injection process and the separation process are listed in Table 2. Each sample was separated twice; one separation was carried out under a low DC voltage to enable a charge-based separation under the linear EK regime, and a second separation process under higher voltage to enable size-based separation under the nonlinear EK regime. The voltages employed in these separations were sufficient to reach the moderate electric field regime, where the velocity of EP_NL_ has a cubic dependence with the electric field ($v_{EP,NL}^{\left(n\right)}$). The required voltages for each distinct separation were identified employing a mathematical model built with COMSOL Multiphysics, model details are in this reference [8]. The next two subsections present the experimental results and the relevant discussion.

### 4.1. Separation of the Binary Particle Mixture of Similar Charge and Different Diameters (5.9 and 11.7 µm)

**Figure 2:**
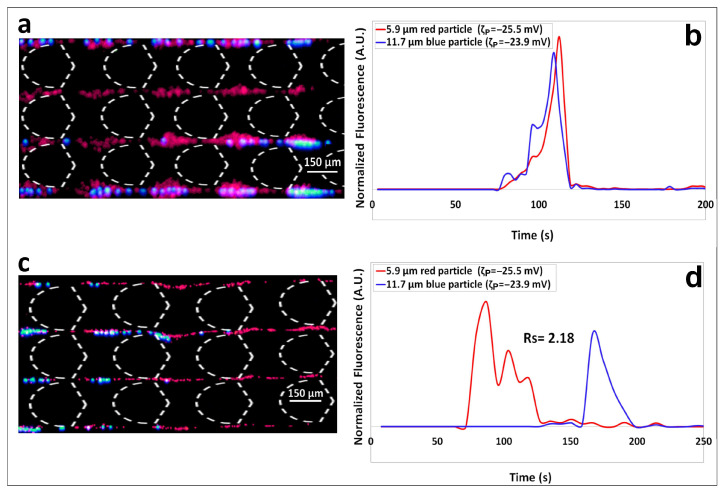
(**a**) Image of the red 5.9 µm and blue 11.7 µm particles as they travel across the post the array. The particles are traveling mixed together, no separation is observed. (**b**) Electropherogram of the charge-based separation showing overlapping particle peaks, confirming that no separation took place under ΔV = 800 V which corresponds to linear EK regime. (**c**) Image of the red 5.9 µm and blue 11.7 µm particles separating into “zones” as they travel across the post the array according to their sizes, the smaller red particles (5.9 µm) are traveling ahead of the larger blue particles (11.7 µm). (**d**) Electropherogram of the size-based separation showing well-defined particle peaks, confirming an effective separation with a *Rs* > 1.5 carried out under ΔV = 1500 V which corresponds to the nonlinear EK regime.

The separation of the mixture containing particle 1 and 2 (5.9 and 11.7 µm diameter, respectively) was carried out twice, under the conditions listed in Table 2. The experimental separation results are included in Figure 2. The first separation, performed under a ΔV = 800 V (E field overall = 151.4 V/cm) between electrodes B–D, illustrates the results of a charge-based separation under the linear EK regime. At these low electric field conditions EP_L_ was the discriminatory EK phenomena that dominated the system, which as seen in Figure 2a,b does not produce a separation. Figure 2a shows the two types of particles as they migrate across the insulating post array, where the two distinct types of particles are migrating mixed together, no separation “zones” are observed. This is further confirmed by the electropherogram in Figure 2b, which shows the two overlapping particle peaks, thus confirming that no separation took place. These results can be easily explained. Under the linear EK regime, EP_L_ is the dominant discriminatory EK phenomenon, which discriminates particles by differences in their surface charge. However, EP_L_ cannot discriminate particles by exploiting size or shape differences [16,17]. Since these two distinct types of particles have very similar surface charge, in terms of their zeta potentials of −25.5 mV and −23.8 mV (difference of 1.7 mV), the small difference in electrical charge is simply not enough to achieve separation. This is an excellent example where nonlinear effects are needed, EP_NL_ is the answer here as it can differentiate particles by their size and shape [14,18,19]. This separation was carried for a second time employing a ΔV = 1500 V (E field overall = 316.1 V/cm) as listed in Table 2. Under these higher electric field conditions EP_NL_ contributed to the discrimination between the two particle types by exploiting the size difference between the two particle types. The size-based separation, which is shown in Figure 2c,d, had excellent results with a separation resolution of *Rs* = 2.18, demonstrating a complete separation. The image in Figure 2c shows the smaller red 5.9 µm particle migrating faster, and the larger blue 11.7 µm particles lagging behind. The electropherogram in Figure 2d shows the smaller particle, exhibiting low EP effects, eluting first, and the larger particle, exhibiting significant EP effects eluted much later. It is important to remember that all particles have negative surface charge, thus, EP effect “delay” particle elution. Good reproducibility between experimental repetitions was obtained for the nonlinear binary separation, with standard deviations in terms of experimental below 16% (Table S3). The confidence interval plots for the electropherograms of the linear and nonlinear separation are reported in Figure S1.

As stated by Khair [14], particle migration under EP_NL_ effects is influence by particle size, this is further confirmed by the magnitude of the mobilities of EP_NL_ measured experimentally in our laboratory [18,25], which as shown in Table 1 lists a much higher value for the $\mu_{EP,NL}^{\left(3\right)}$ magnitude of the 11.7 µm particles. This results are in agreement with Khair [14], Dukhin [23] and with experimental results previously obtained by our group [18]. Larger particles exhibit stronger EP_NL_ effects and have greater magnitudes of EP_NL_ mobilities since the larger particle size leads to an increase in the conductive-diffuse layer of the electrical double layer (EDL) around the particle, which in turn increases the polarization charge in the EDL. This binary microparticle separation is an excellent example of how elution order can be manipulated by simply engaging EP_NL_ effects by tuning the applied voltage. The first separation under low voltage conditions had the two particle types eluting together; this was changed in the second separation, performed and higher voltage conditions, where the larger particle eluted much later than the smaller particle in the mixture.

### 4.2. Separation of the Two Tertiary Particle Mixtures of Particles of Different Diameters (4.1, 7.4 and 11.7 µm) and (2.4, 5.7 and 11.7 µm)

**Figure 3:**
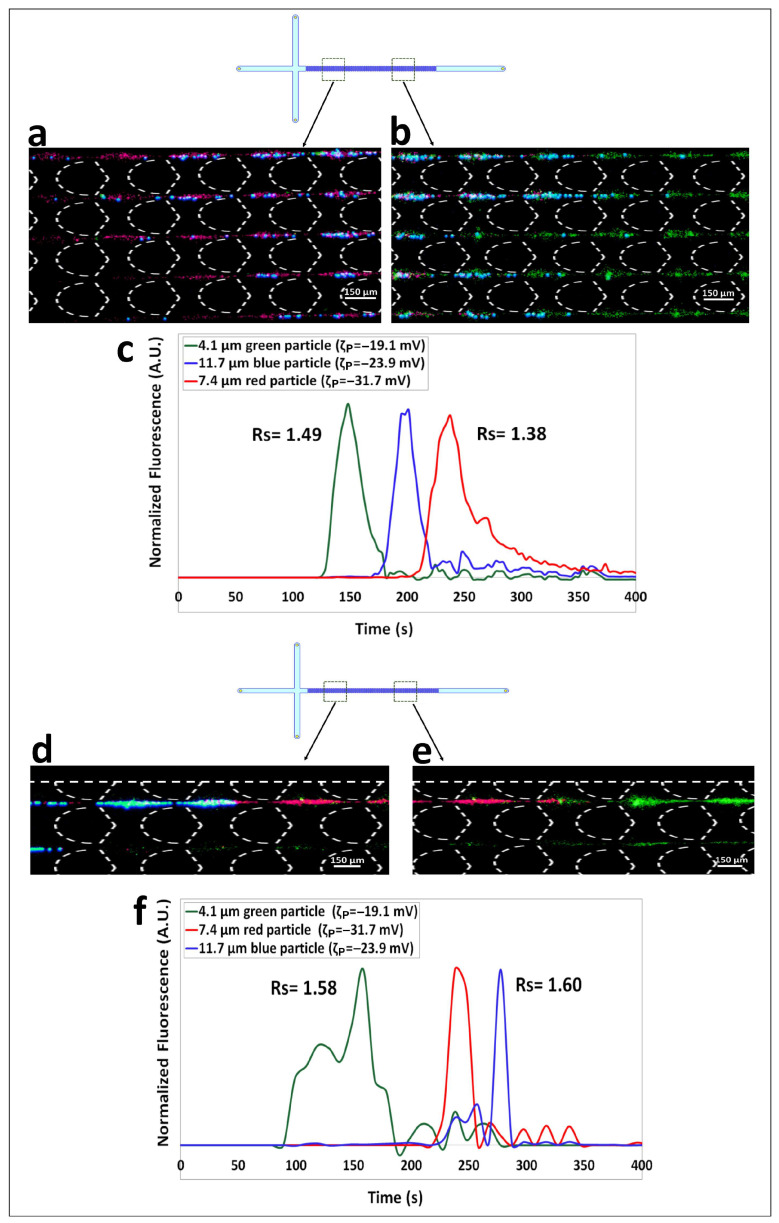
(**a**,**b**) Images of the green 4.1 µm, red 7.4 µm, and blue 11.7 µm particles as they travel across the post the array according to their electrical charge. In this charge-based separation the green particles are traveling ahead, followed by the blue particles, with the red particles being the slowest. (**c**) Electropherogram of the size-based separation showing three well defined particle peaks where both *Rs* values are below 1.5. This electropherogram was obtained with ΔV = 400 V which corresponds to linear EK regime. (**d**,**e**) Images of the green 4.1 µm, red 7.4 µm and blue 11.7 µm particles separating into “zones” according to their sizes, the smallest particle (green, 4.1 µm) is the fastest and the largest particle (blue, 11.7 µm) is the slowest. (**f**) Electropherogram of the tertiary size-based separation showing well defined peaks with both *Rs* values above 1.5 carried out under ΔV = 1500 V which corresponds to the nonlinear EK regime.

The two tertiary separations in this work employed two distinct mixtures of particle of distinct size (ranging from 2.4 to 11.7 µm in diameter) with zeta potentials between −19 to −34 mV (particles ID # 2, 3, 4 and # 2, 5, 6 in Table 1). First, separation of particles with diameter of 4.1, 7.4 and 11.7 µm was carried out twice, under the linear EK regime and under the nonlinear EK regime, and the results are included in Figure 3. Under the linear regime, with EP_L_ being the discriminatory separation mechanism, particles are separated according to their electrical charge, which produced an elution according to increasing magnitude of the negative particle zeta potential. The results from the linear EK regime charge-based separation are shown in Figure 3a–c. Under an ΔV = 400 V (E field overall = 57.2 V/cm) between electrodes B–D. The three types of particles migrated as shown in Figure 3a,b: green 4.1 µm particles are the fastest since their zeta potential magnitude is the lowest ($\zeta_{P}$ = −19.1 mV), they are followed by the blue 11. 4 µm particles ($\zeta_{P}$ = −23.9 mV), with the red 7.4 µm particles being the slowest ($\zeta_{P}$ = −31.7 mV). These results are further confirmed by the electropherogram in Figure 3c depicting three well-defined particle peaks for this charge-based separation, with separation resolutions of Rs_1,2_ = 1.49 and Rs_2,3_ = 1.38 between peaks 1–2 and peaks 2–3, respectively. Since neither of the two *Rs* values reached 1.5, which is considered a complete separation, it was attractive to investigate the performance that can be obtained by carrying out the separation under the nonlinear EK regime. As discussed, EP_L_ can only separate particle by exploiting differences in electrical charge [16,17]. Similarly to the separation of the binary mixture, EP_NL_ is the answer to achieve complete separation by exploiting the size differences between these three particles [14,18,19]. The tertiary separation was carried out again, this time under ΔV = 1500 V (E field overall = 316.1 V/cm) as listed in Table 2, to enable a size-based separation, and the results are shown in Figure 3d–f. The migration order of the particles (Figure 3d,e), as traveled along the post array, was determined by particle size, the smallest particles (red, 7.4 µm) were the fastest, followed by the medium size particles (green, 4.1 µm), with the largest particles (blue, 11.7 µm) being the slowest. The electropherogram in Figure 3f confirms these results, as the particles peaks eluted according to increasing particle size. This elution order is as expected from the magnitude of $\mu_{EP,NL}^{\left(3\right)}$ of each particle type, the larger the particle, the larger the magnitude of its $\mu_{EP,NL}^{\left(3\right)}$. Larger particles exhibit stronger EP_NL_ effects due to the increased volume of their conductive-diffuse layer [23]. These results provide another example where the use of EP_NL_ effects alter the elution order of the particles in the separation and also improves the separations resolutions (both *Rs* values > 1.5) when compared to the performance obtained with EP_L_ (both *Rs* values < 1.5). Good reproducibility between experimental repetitions was obtained for the linear and nonlinear tertiary separation, with standard deviations in terms of experimental below 22% (Table S3) in both cases. The confidence interval plots for the electropherograms of the linear and nonlinear separations are reported in Figure S2.

**Figure 4:**
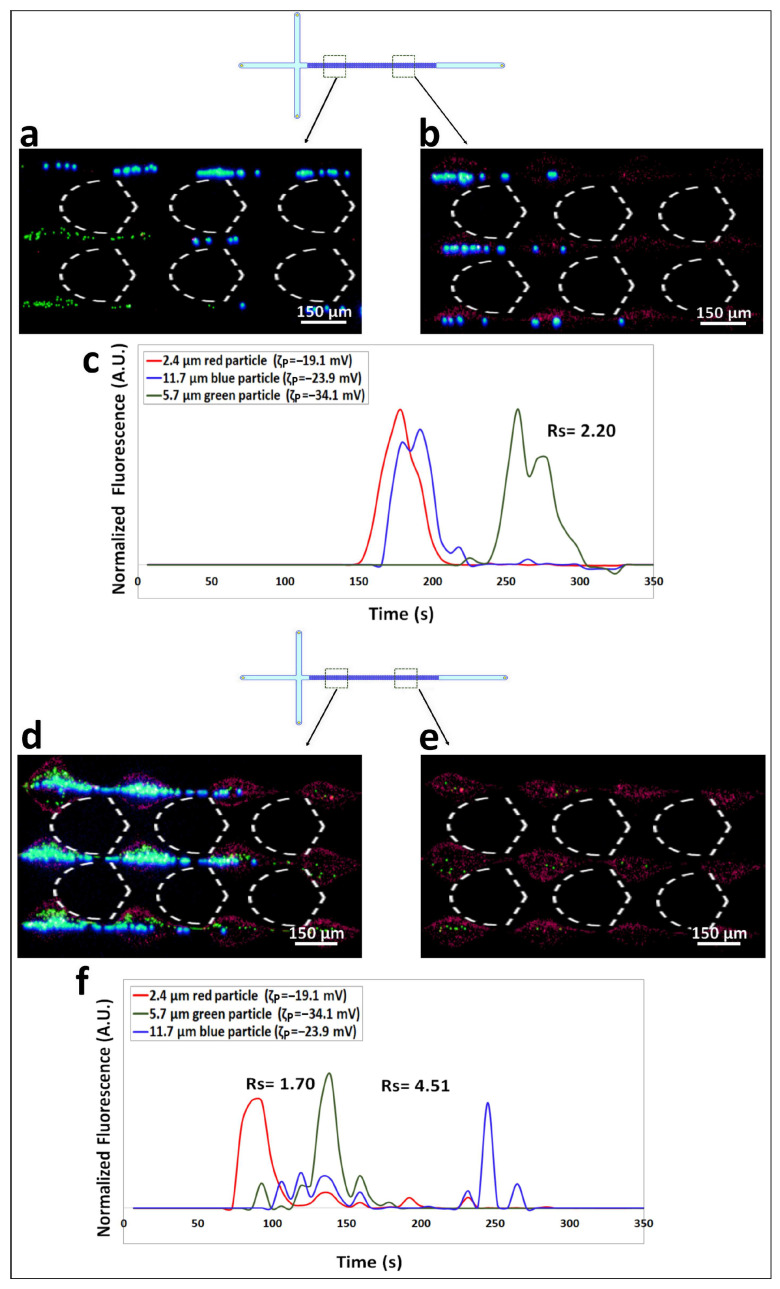
(**a**,**b**) Images of the red 2.4 µm, blue 11.7 µm, and green 5.7 µm particles as they travel across the post the array according to their electrical charge. In this charge-based separation the red and blue particles are traveling ahead, almost eluting at the same time, with the green particles being the slowest. (**c**) Electropherogram of the charge-based separation showing three peaks where no separation is observed for red and blue particles. This electropherogram was obtained with ΔV = 800 V which corresponds to linear EK regime. (**d**,**e**) Images of the red 2.4 µm, green 5.7 µm and blue 11.7 µm particles separating into “zones” according to their sizes, the smallest particle (red, 2.4 µm) is the fastest and the largest particle (blue, 11.7 µm) is the slowest. (**f**) Electropherogram of the tertiary size-based separation showing well defined peaks with both *Rs* values above 1.5 carried out under ΔV = 1500 V which corresponds to the nonlinear EK regime.

To further demonstrate the effectiveness of the proposed method for altering the elution order in a tertiary separation of particles, an additional tertiary separation was performed employing a different combination of particles with diameters of 2.4, 5.7, and 11.7 µm under the linear and the nonlinear regimes. The results of this separation are reported in Figure 4 and Table S3. The findings from this second tertiary separation corroborated those obtained with the initial tertiary separation, further demonstrating that distinct elution orders can be obtained by switching from the linear to the nonlinear EK regime. As shown in Figure 4a–c, the separation of particles in a linear regime under ΔV = 800 V (E field overall = 151.4 V/cm) produces no separation between red (2.4 µm) and blue (11.7 µm) particles, making them elute almost at the same time as EP_L_ is dominant. However, by performing the separation under the higher applied voltage of ΔV = 1500 V (E field overall = 316.1 V/cm), which corresponds to the nonlinear regime, the smallest particle (red, 2.4 µm) elutes first, followed by green particles (5.7 µm), and finally, the largest particle (blue, 11.7 µm) eluted last. Achieving separation resolutions of Rs_5,6_ = 1.70 between red and green particles, and Rs_6,2_ = 4.51 between green and blue particles, underscores the applicability of this method for separating tertiary particle mixtures. The confidence interval plots for the linear and nonlinear separations are reported in Figure S3.

## 5. Conclusions

Migration order is a major aspect in all migration-based analytical separation techniques, such as chromatography and capillary electrophoretic methods. Significant research has been conducted to identify strategies for manipulating the migration or elution order of the analytes in migration-based separations. However, there is a lack of similar methods that can alter elution order in separation of micron-sized particles. This study presents a strategy for manipulating the migration order of microparticles in insulator-based electrokinetic devices. The strategy was illustrated by performing two distinct microparticle separations (a binary separation and a tertiary separation) twice: one trial under the linear electrokinetic regime and trial under the nonlinear electrokinetic regime. Under the linear electrokinetic regime, the discriminatory mechanism is linear electrophoresis, whereas under the nonlinear electrokinetic regime, the discriminatory mechanism is nonlinear electrophoresis. A main difference between linear and nonlinear electrophoresis, is that linear electrophoresis can only separate analytes by charge differences, while nonlinear electrophoresis separates analytes by size differences.

The results illustrated that a distinct elution order is obtained under each electrokinetic regime. Under the linear electrokinetic regime particle migration order is dictated by the particles’ electrical charge, while under the nonlinear electrokinetic regime, particle migration order is dictated by the particle’s size. Furthermore, for both separation, binary and tertiary, the separation performance in terms of separation resolution (Rs) was better under the nonlinear electrokinetic regime. The results from this study demonstrate that manipulation of the migration order of analytes is possible in insulator-based electrokinetic systems by switching from linear to nonlinear electrokinetic regimes. These findings show new possibilities for the electrokinetic-based separation of micron-sized particles such as microorganisms and mammalian cells. This study is the very first report on the use of nonlinear electrophoresis altering the elution order in an electrophoresis-based separation.

## Figures and Tables

**Table 1:** Characteristics of the microparticles employed in this study.

Particle ID	Color	Diam. (μm)	$\zeta_{P}$ (mV)	$\mu_{EP,L}$ × 10^−8^ (m^2^V^−1^s^−1^)	$E\;\mathrm{Used}\;\mathrm{for}\;\mu_{EP,NL}^{\left(3\right)}$ Estimation (V/cm) *	$\mu_{EP,NL}^{\left(3\right)}$ × 10^−18^ (m^4^V^−3^s^−1^)
1	Red	5.9 ± 0.3	−25.5 ± 4.2	−2.0 ± 0.3	150	−8.6 ± 7.1
2	Blue	11.7 ± 0.2	−23.9 ± 1.1	−1.9 ± 0.1	100	−23.2 ± 16.7
3	Green	4.1 ± 0.3	−19.1 ± 3.2	−1.5 ± 0.1	350	−2.1 ± 1.8
4	Red	7.4 ± 0.3	−31.8 ± 1.8	−2.5 ± 0.1	100	−7.3 ± 5.3
5	Red	2.4 ± 0.1	−19.1 ± 2.2	−1.5 ± 0.1	400	−3.2 ± 1.5
6	Green	5.7 ± 0.2	−34.1 ± 3.7	−2.7 ± 0.2	150	−16.1 ± 1.2

* Table S1 in the Supplementary Materials contains the values of the parameters used to determine the electric field regime as moderate (E^3^) under the current operating conditions. Table S2 contains the particle concentration information.

**Table 2:** Applied voltage conditions used for iEK injection and microparticle separations.

Separation Description	Step	Run Time (s)	Applied Voltage (V)			
			A	B	C	D
Binary linear EK regime (Figure 2b)	Loading	20	2500	100	0	1000
	Gating	1	1500	2500	1500	0
	Injection & Separation	550	200	800	200	0
Binary nonlinear EK regime (Figure 2d)	Loading	20	2500	100	0	1000
	Gating	1	1500	2500	1500	0
	Injection & Separation	500	200	1500	200	0
Tertiary linear EK regime (Figure 3c)	Loading	30	1500	100	0	1000
	Gating	6	1500	1500	1500	0
	Injection & Separation	500	200	400	200	0
Tertiary nonlinear EK regime (Figure 3f)	Loading	20	2500	100	0	1000
	Gating	1	1500	2500	1500	0
	Injection & Separation	500	200	1500	200	0
Tertiary linear EK regime (Figure 4c)	Loading	10	1500	100	0	1000
	Gating	1	1500	2500	1500	0
	Injection & Separation	350	200	800	200	0
Tertiary nonlinear EK regime (Figure 4f)	Loading	10	2500	100	0	1000
	Gating	1	1500	2500	1500	0
	Injection & Separation	350	200	1500	200	0

## Data Availability

The data presented in this study are available on request from the corresponding author.

## Supplementary Materials

The following supporting information can be downloaded at: https://www.mdpi.com/article/10.3390/bios14030119/s1, Table S1: Parameters employed to determine electric the field regime of electrophoretic particle migration; Table S2: Concentration of the particle and cell sample suspensions employed in this study; Table S3: Experimental retention of each particle in each experimental repetition performed, including the standard deviation between experimental repetitions. Figures S1–S3: Confidence interval plots for linear and nonlinear separations.
