# Supplementary material for: Switching Separation Migration Order by Switching Electrokinetic Regime in Electrokinetic Microsystems
Source: Biosensors (Basel). 2024 Feb 22;14(3):119. doi: 10.3390/bios14030119 (PMC10968109; doi:10.3390/bios14030119)
Supplement: Supplementary file 1 [file biosensors-14-00119-s001.zip › biosensors-2814852-supplementary.pdf]

Supplementary Materials

Switching separation migration order by switching electrokinetic regime in electrokinetic microsystems

Alaleh Vaghef-Koodehi and Blanca H. Lapizco-Encinas *

Microscale Bioseparations Laboratory and Biomedical Engineering Department, Rochester Institute of Technology, 160 Lomb Memorial Drive, Rochester, New York, 14623, United States.

* Correspondence: bhlbme@rit.edu

Contents

PARAMETERS USED TO DETERMINE THE APPROPRIATE ELECTRIC FIELD REGIME OF ELECTROPHORETIC PARTICLE MIGRATION: TABLE S1 2

PARTICLE CONCENTRATION INFORMATION: INCLUDES TABLE S2 2

REPRODUCIBILITY BETWEEN EXPERIMENTAL REPETITIONS: INCLUDES TABLE S3 3

CONFIDENCE INTERVAL PLOTS FOR LINEAR AND NONLINEAR SEPARATIONS: INCLUDES FIGURE S1 TO FIGURE S3 3

Parameters used to determine the appropriate electric field regime of electrophoretic particle migration: Table S1

Three dimensionless parameters are employed to assess the electric field regime of electrophoretic particle migration, these parameters are: the dimensionless applied field strength coefficient (β), the Peclet number (*Pe*) and the Dukhin number (*Du*). The expressions used to describe these parameters in this study are

$$\beta = \frac{Ea}{\varphi} \tag{S1}$$

$$Pe = \frac{a|(\mathbf{v}_{EP,L} + \mathbf{v}_{EP,NL})|}{D} \tag{S2}$$

$$Du = \frac{K^{\sigma}}{K_m a} \tag{S3}$$

where E is applied electric field, a is the particle radius, and φ is the thermal voltage (~25 mV), D is the diffusion coefficient, K^{σ} and K^{m} are the surface and bulk conductivity of the medium, respectively.

Table S1. Parameters employed to determine electric the field regime of electrophoretic particle migration.

Separation ID	Particle ID	Diameter (µm)	ζ_P (mV)	E for $\mu_{EP}^{(3)}$ estimation (V/cm)	β	*Pe*	*Du*	Electric field regime
1	1	5.9 ± 0.3	-25.5 ± 4.2	150	2.0	0.6	0.02	Moderate ($\mathbf{E}^3$)
	2	11.7 ± 0.2	-23.8 ± 1.1	100	2.7	0.8	0.01	Moderate ($\mathbf{E}^3$)
2	3	4.1 ± 0.3	-19.1 ± 3.2	350	2.7	0.7	0.02	Moderate ($\mathbf{E}^3$)
	4	7.4 ± 0.3	-31.8 ± 1.8	100	1.7	0.6	0.02	Moderate ($\mathbf{E}^3$)
	2	11.7 ± 0.2	-23.8 ± 1.1	100	2.7	0.7	0.01	Moderate ($\mathbf{E}^3$)
3	5	7.4 ± 0.3	-31.8 ± 1.8	100	2.0	0.8	0.03	Moderate ($\mathbf{E}^3$)
	6	2.4 ± 0.1	-19.1 ± 2.2	400	2.1	0.6	0.04	Moderate ($\mathbf{E}^3$)
	2	11.7 ± 0.2	-23.8 ± 1.1	100	2.7	0.7	0.01	Moderate ($\mathbf{E}^3$)

Particle concentration information: Includes Table S2

Particle suspensions to be used in experimentation were prepared by dilution the stock solution into the suspending medium, the concentration of each particle suspension used in this study is included below:

Table S2. Concentration of the particle and cell sample suspensions employed in this study.

Separation ID	Particle ID	Diameter (µm)	Concentration x 10^7 (# particles/mL)
1	1	5.9 ± 0.3	1.8
	2	11.7 ± 0.2	0.6
2	3	4.1 ± 0.3	6.9
	4	7.4 ± 0.3	3.2
	2	11.7 ± 0.2	1.6
3	5	2.4 ± 01	19.6
	6	5.7 ± 0.2	3.4
	2	11.7 ± 0.2	0.3

Reproducibility between experimental repetitions: includes Table S3

Each experimental separation was repeated at least three times, the values of the experimental retention times of three distinct repetition of each separation are listed in **Table S3.**

Table S3. Experimental retention of each particle in each experimental repetition performed, including the standard deviation between experimental repetitions. The results from the binary linear separation are not included in this table, since the peaks overlapped in each one of the repetitions.

Separation Description	Particles	$t_{R,e}$ Repetition 1 (s)	$t_{R,e}$ Repetition 2 (s)	$t_{R,e}$ Repetition 3 (s)	STDEV (%)
Binary nonlinear EK regime	Red, 5.9 µm	87	98	78	11%
	Blue, 11.7 µm	174	160	146	9%
Tertiary linear EK regime	Green, 4.1 µm	148	184	142	14%
	Blue, 11.7 µm	200	248	187	15%
	Red, 7.4 µm	260	342	270	15%
Tertiary nonlinear EK regime	Green, 4.1 µm	158	121	153	14%
	Red, 7.4 µm	237	154	193	21%
	Blue, 11.7 µm	277	205	266	16%
Tertiary linear EK regime	Red, 2.4 µm	178	140	112	23%
	Blue, 11.7 µm	191	152	121	23%
	Green, 5.7 µm	257	224	195	14%
Tertiary nonlinear EK regime	Red, 2.4 µm	92	81	87	6%
	Green, 5.7 µm	138	180	138	16%
	Blue, 11.7 µm	244	261	269	5%

Confidence interval plots for linear and nonlinear separations: includes Figure S1 to Figure S3

Confidence interval plots are plotted for all linear and nonlinear separation which include Figure S1, Figure S2, and Figure S3. In all cases the confidence plots for nonlinear separation produce more well-defined peaks compare to linear separations.

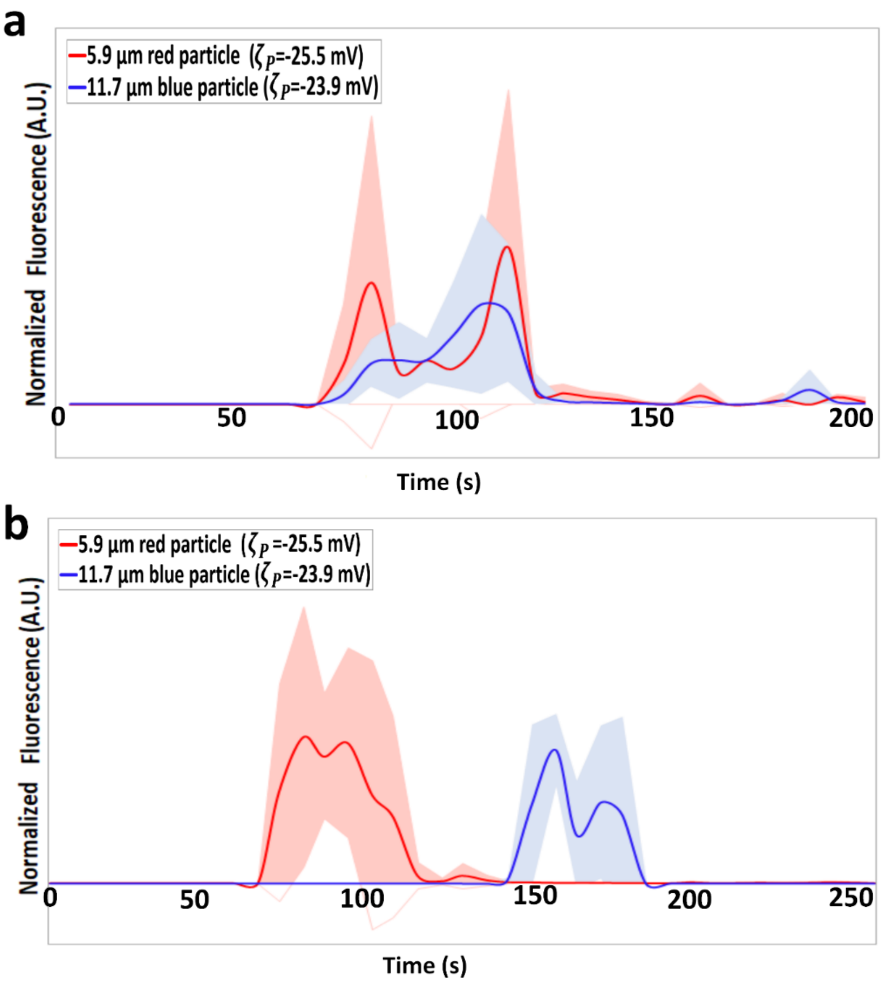

Figure S1. Confidence interval plots for the binary separation (Separation ID= 1). (a) Confidence interval plots for the repetitions of the charge-based separation showing overlapping particle peaks, confirming that no separation took place under ΔV = 800 V which corresponds to linear EK regime. (b) Confidence interval plots for the repetitions of the size-based separation under ΔV = 1500 V which corresponds to nonlinear EK regime.

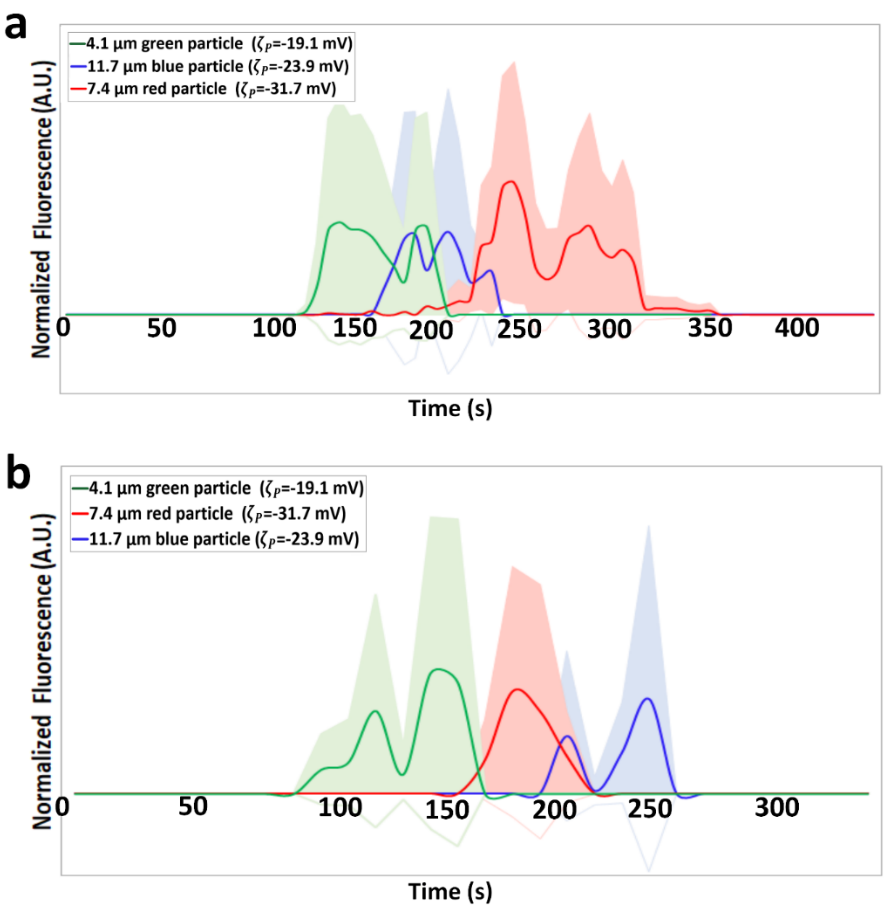

Figure S2. Confidence interval plots for the first tertiary separation. (a) Confidence interval plots for the repetitions of the charge-based separation showing overlapping particle peaks under ΔV = 400 V which corresponds to linear EK regime. (b) Confidence interval plots for the repetitions of the size-based separation under ΔV = 1500 V which corresponds to nonlinear EK regime, showing less overlapped peaks compared to linear confidence intervals.

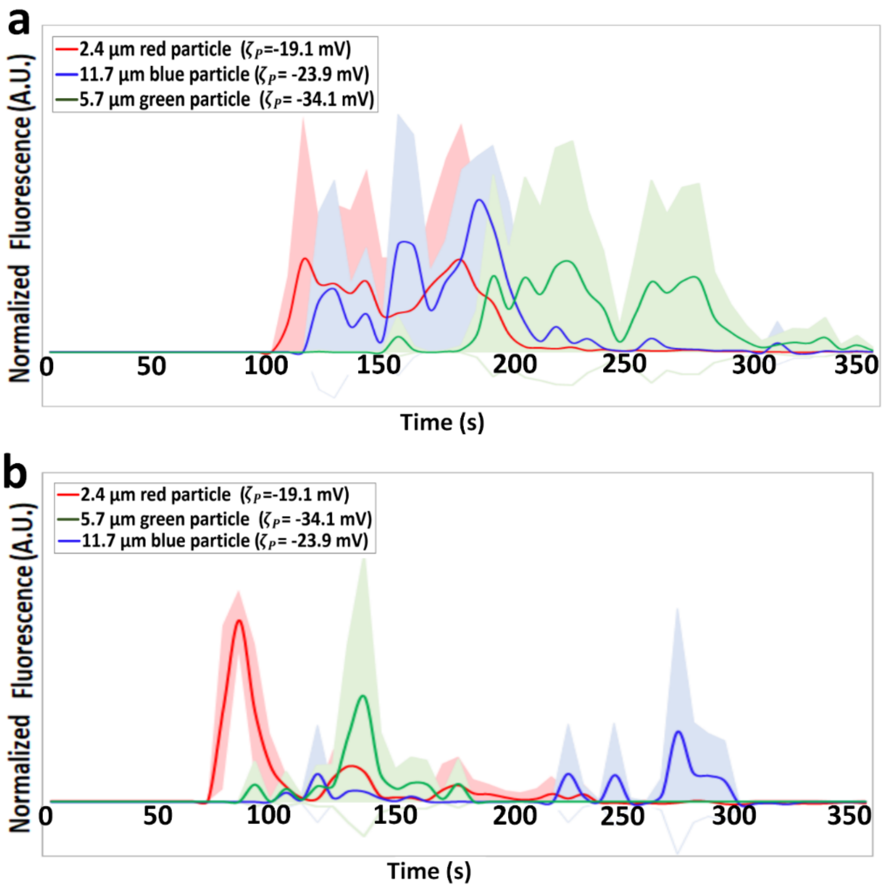

Figure S3. Confidence interval plots for the second tertiary separation. (a) Confidence interval plots for the repetitions of the charge-based separation showing overlapping particle peaks under ΔV = 800 V which corresponds to linear EK regime. (b) Confidence interval plots for the repetitions of the size-based separation under ΔV = 1500 V which corresponds to nonlinear EK regime, demonstrating well defined peaks compare to linear repetitions.
